# Bildgebung in der gelenkerhaltenden Hüftchirurgie

**DOI:** 10.1007/s00117-022-00973-0

**Published:** 2022-03-03

**Authors:** M. K. Meier, T. D. Lerch, M. S. Hanke, M. Tannast, S. D. Steppacher, F. Schmaranzer

**Affiliations:** 1 Department für Orthopädische Chirurgie und Traumatologie, Inselspital, Universitätsspital Bern, Universität Bern, Bern, Schweiz; 2 Department für Diagnostische, Interventionelle und Pädiatrische Radiologie, Inselspital, Universitätsspital Bern, Universität Bern, Bern, Schweiz; 3 Department für Orthopädische Chirurgie und Traumatologie, HFR Fribourg – Kantonsspital, Universität Fribourg, Fribourg, Schweiz

**Keywords:** Hüftgelenk, Femoroazetabuläres Impingement, Hüftdysplasie, Magnetresonanztomographie, Arthroskopie, Hip joint, Femoroacetabular impingement, Hip dislocation, Magnetic resonance imaging, Arthroscopy

## Abstract

Instabilität und Impingement stellen die Hauptpathomechanismen dar, die bereits bei jungen Patienten durch erhöhten mechanischen Stress zu chondrolabralen Schäden, schmerzhafter Bewegungseinschränkung und frühzeitiger Coxarthrose führen können. Ziele der gelenkerhaltenden Chirurgie an der Hüfte sind die Korrektur der knöchernen Deformitäten und chondrolabraler Schäden sowie die Wiederherstellung der Gelenkfunktion. Voraussetzung dafür ist die Identifikation der ursächlichen Pathologien an der Hüfte, welche zudem in Kombination auftreten können. Die dezidierte Röntgen- und Magnetresonanzbildgebung der knöchernen Morphologie und der degenerativen Gelenkbinnenläsionen liefern einen essenziellen Beitrag für die Behandlungsindikation und die Behandlungsplanung. Der vorliegende Artikel soll einen kurzen Überblick über die Hüftdeformitäten mit deren Prävalenz, Pathomechanismus und indizierter Therapie sowie detaillierte Empfehlungen über die spezifische radiologische Abklärung geben.

## Abstract (en)

Instability and impingement of the hip are the main pathomechanisms that can lead to chondrolabral damage, painful restriction of motion and early hip osteoarthritis due to increased mechanical stress, even in young patients. The goals of joint-preserving hip surgery are correction of the osseous deformities and chondrolabral damage as well as restoration of joint function. A prerequisite for successful surgery is the identification of the underlying hip pathologies, which can also occur in combination. Dedicated radiographic and magnetic resonance (MR) imaging of osseous morphology and the internal degenerative joint lesions play an essential role for the treatment indications and surgical treatment planning. This article provides a concise overview of the prevalence, pathomechanisms and indicated treatment of hip deformities as well as detailed recommendations on the specific radiological clarification.

## Lernziele

Nach Absolvieren dieser Fortbildungseinheit kennen Sie …

- die knöchernen Morphologien, die zu einem Impingement und/oder zur Instabilität der Hüfte führen.
- das Spektrum der hüftgelenkerhaltenden Chirurgie.
- die aktuellen Empfehlungen zur diagnostischen Abklärung vor einem gelenkerhaltenden Hüfteingriff.
- den Stellenwert der jeweiligen Modalitäten für die Therapieplanung.

## Einleitung

Hüftdeformitäten können bei jungen, aktiven Patienten zu schmerzbedingten und funktionellen Einschränkungen im Alltag sowie zu vorzeitiger Arthrose führen. Nach dem Pathomechanismus können 2 Hauptdeformitäten unterschieden werden:

- Hüftdysplasie, bei der es durch mangelnde azetabuläre Überdachung zu einer statischen Instabilität kommt;
- femoroazetabuläres Impingement (FAI), bei dem es zu einem frühzeitigen dynamischen knöchernen Konflikt zwischen proximalem Femur und Becken kommt.

Bei beiden Deformitäten führt die veränderte Biomechanik zu erhöhtem intraartikulären Stress des Gelenkknorpels und des azetabulären Labrums und unter Umständen zur vorzeitigen **Coxarthrose**

Coxarthrose

[1]. Typischerweise treten Hüftdeformitäten nicht in Reinform, sondern in Kombination auf und können sich gegenseitig verstärken oder auch kompensieren (Tab. 1). Die hüftgelenkerhaltende Chirurgie hat das Ziel, diese Deformitäten zu korrigieren, eine normale Gelenkfunktion wiederherzustellen und idealerweise das Fortschreiten einer derartigen „Präarthrose“ aufzuhalten. Das chirurgische Spektrum umfasst die offene/arthroskopische Schenkelhalstaillierung und Pfannenrandtrimmung zur Behandlung von Cam- und fokalen Pincer-Deformitäten sowie Femur- und Beckenosteotomien zur Korrektur der Hüftdysplasie und von Rotationsfehlstellungen. Aufgrund der hohen Prävalenz von asymptomatischen **Impingement-Morphologien**

**Figure 1:**
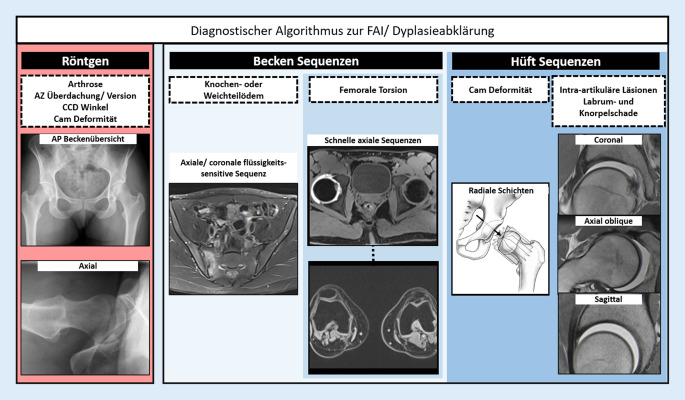

**Figure 2:**
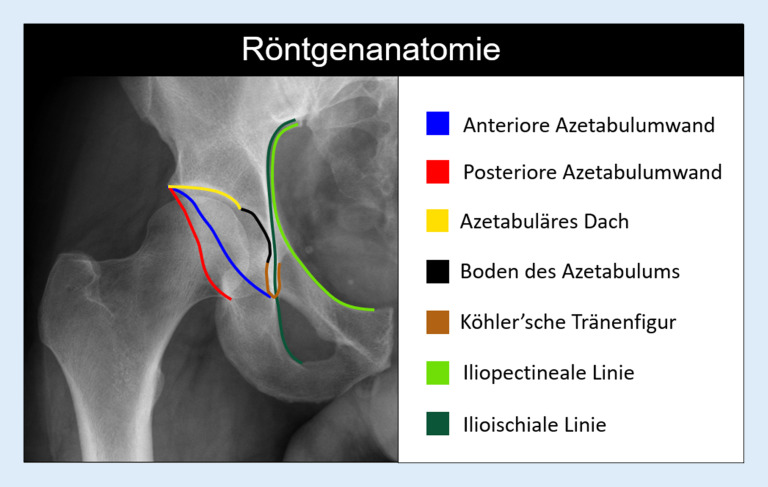

**Figure 3:**
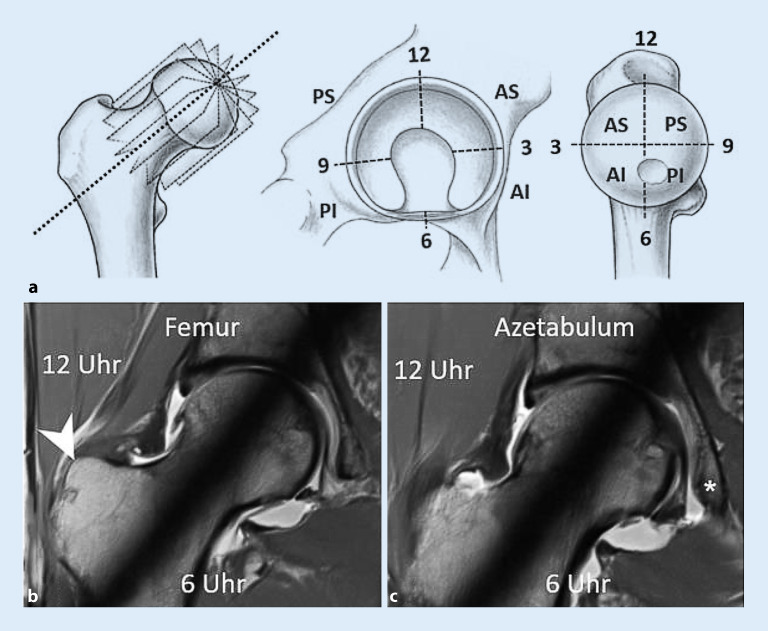

Impingement-Morphologien

sollten diese nur mit entsprechender klinischer Symptomatik als solche bezeichnet und weiter abgeklärt werden. Die präoperative Bildgebung mittels Röntgen, Magnetresonanztomographie (MRT) und Computertomographie (CT) ist essenziell für die diagnostische Abklärung der vorliegenden knöchernen Deformitäten sowie zur Beurteilung von Schäden an den Gelenkbinnenstrukturen. Ziel dieses Artikels ist es, die aktuellen Empfehlungen zum diagnostischen Protokoll sowie zur Klassifizierung der knöchernen Deformitäten und der chondrolabralen Schäden, basierend auf dem 2020 erschienenen internationalen Lissaboner Konsensusstatement der European Society of Musculoskeletal Radiology (ESSR), zusammenzufassen (Abb. 1; [2, 3]).

**Table 1:** 

	Impingement				Instabilität	
	Anterior		Posterior (ischiofemoral)		Dynamisch	Statisch
	Intraartikulär	Extraartikulär	Trochanter major	Trochanter minor		
Dysplasie	–	–	–	–	+	+++
Erhöhte azetabuläre Anteversion	–	–	+	+	+	++
Azetabuläre Retroversion	+++	++	–	–	–	–
Azetabuläre Mehrüberdachung	+++	+	–	–	+	–
Cam-Deformität	+++	+	–	–	–	–
Erhöhte femorale Antetorsion	–	–	++	+++	+++	+
Femorale Retrotorsion	+++	++	–	–	+	–
Coxa vara	+	+	+	–	–	–
Coxa valga	+	–	++	+++	++	+

### Merke

Die Hüftgelenkdysplasie und das femoroazetabuläre Impingement sind die Hauptpathomechnismen bei jungen Patienten mit Hüftschmerzen.

## Modalitäten

### Projektionsradiographie

Das konventionelle Röntgen bildet den Grundstein der bildgebenden Diagnostik des Hüftgelenks (Abb. 1) und besteht standardmäßig aus 2 Aufnahmen:

- Beckenübersichtsröntgen im anteroposterioren (a.p.) Strahlengang,
- axiales Bild des proximalen Femurs.

Die standardisierte Akquisition der Beckenübersichtsaufnahme im Liegen und mit 15° innenrotierten Beinen, mit einem **Film-Fokus-Abstand**

Film-Fokus-Abstand

von 1,2 m sowie Zentrierung auf den Schnittpunkt einer Tangente zwischen Spina iliaca anterior superior und Symphysenoberkante ist entscheidend, um eine akkurate Projektion der Beckenanatomie zu gewährleisten. Diese Projektion dient der Beurteilung der Beckenorientierung sowie der azetabulären Version und Überdachung anhand projektionsradiographischer Kennlinien, deren Kenntnis Voraussetzung zur Beurteilung der Hüftgelenkmorphologie ist (Abb. 2). Basierend auf der **Tönnis-Klassifikation**

Tönnis-Klassifikation,

die neben anderen Arthrosezeichen v. a. auf der Beurteilung der Gelenkspaltverschmälerung beruht (Tönnis-Grad 0/1/2/3 = keine/wenig/mäßige/ausgeprägte Gelenkspaltverschmälerung), erfolgt eine erste Stratifizierung in Bezug auf die Prognose nach einem gelenkerhaltenden Eingriff [4]. Diese nimmt mit zunehmender Schwere des Arthrosegrades (> Tönnis 1) und mit zunehmendem Alter (> 40 Jahre) drastisch ab [5, 6]. Bei älteren Patienten mit manifester Arthrose ist die Implantation einer **Hüfttotalendoprothese**

Hüfttotalendoprothese

die Therapie der Wahl und eine auf die Hüfte fokussierte MRT zur Beurteilung der Gelenkdegeneration weitgehend obsolet. Die Beckenübersichtsaufnahme erlaubt nur die Darstellung von Schenkelhalstaillierungsstörungen, die nach kraniolateral reichen (sog. **Pistol-grip-Deformitäten**

Pistol-grip-Deformitäten).

Zur besseren Visualisierung der typischerweise ventrosuperioren **Cam-Deformität**

Cam-Deformität

bedarf es einer zusätzlichen axialen Aufnahme. Von der Vielzahl an beschriebenen axialen Aufnahmen (Cross-table‑, Lauenstein-Projektion etc.) eignet sich die modifizierte **Dunn-Projektion**

Dunn-Projektion

(45° Hüftflexion in maximaler Hüftabduktion, Zentralstrahl auf den Femurkopf ausgerichtet) am besten zur initialen und postoperativen Beurteilung der Schenkelhalstaillierung [2].

#### Merke

Basis der radiologischen Diagnostik sind das Beckenübersichtsröntgen sowie eine axiale Aufnahme.

### Magnetresonanztomographie der Hüfte

Die MRT stellt die zweite Ebene in der diagnostischen Pyramide zur Abklärung eines hüftgelenkerhaltenden Eingriffs dar und sollte mindestens bei 1,5 T erfolgen. Zum groben Ausschluss von entzündlichen periartikulären Veränderungen z. B. am Iliosakralgelenk oder an der Symphysis pubica sind routinemäßig flüssigkeitssensitive Sequenzen (**TIRM [„turbo inversion recovery magnitude“]**

TIRM („turbo inversion recovery magnitude“)

/fettgesättigte T2-gewichtete [T2w] TSE [„turbo spin echo“]) des Beckens in koronaler oder axialer Orientierung zu akquirieren [2, 7]. Im Anschluss sollten aufeinanderfolgende, schnelle axiale Sequenzen über Becken und Knie (z. B. dreidimensionale [3-D] T1w-Dixon-**VIBE [„volumetric interpolated breath-hold examination“]**

VIBE („volumetric interpolated breath-hold examination“))

zur Bestimmung der Femurtorsion akquiriert werden [2, 8]. Zudem eignen sich diese Sequenzen auch zur Detektion von typischen Muskelödemen in der ischiofemoralen Loge, welche mit einem posterioren extraartikulären Impingement assoziiert sind [9]. Zur Darstellung chondrolabraler Schäden bedarf es hüftzentrierter, hochaufgelöster Sequenzen in koronaler, sagittaler und „axial oblique“ Orientierung. Die direkte MR-Arthrographie gilt hier als Methode der Wahl zur Detektion intraartikulärer Läsionen, obgleich mit einem hochaufgelösten nativen Protokoll bei 3 T auch eine hohe diagnostische Genauigkeit erzielt werden kann [4]. Radiäre Sequenzen, welche orthogonal um die Schenkelhalsachse rotieren, sind hilfreich zur Beurteilung und Lokalisation der Cam-Deformität (Abb. 3). Die hüftzentrierten Sequenzen können entweder direkt mittels 2‑D-TSE/FSE(„fast spin echo“)-Sequenzen akquiriert oder aus einer 3‑D-Sequenz rekonstruiert werden [4]. Hochaufgelöste, isotrope 3‑D-Sequenzen (z. B. 3‑D-T2w-**TRUFI [„true fast imaging with steady precession“])**

TRUFI („true fast imaging with steady precession“)

erlauben zudem aufgrund der dünneren Schichtführung oftmals eine genauere Charakterisierung chondrolabraler Schäden [10].

#### Merke

Die direkte MR-Arthrographie ist der Goldstandard zur Detektion chondrolabraler Schäden.

### Computertomographie der Hüfte

Als Alternative zur MR-Arthrographie erlaubt die CT-Arthrographie mit einer hohen diagnostischen Genauigkeit die Detektion chondrolabraler Läsionen. Vorteil der CT ist neben der flächendecken Verfügbarkeit die Möglichkeit eines schnellen und unkomplizierten 3‑D-Volume-Renderings im PACS („picture archiving and communication system“; [4]). CT-basierte 3‑D-Modelle können zudem auch zur dynamischen Impingement-Simulation verwendet werden, was speziell bei Patienten mit komplexen Deformitäten und Verdacht auf ein extraartikuläres Impingement hilfreich zur Operationsplanung ist [11, 12]. Fortschritte in der Gerätetechnik, wie z. B. die Verwendung eines **Zinnfilters**

Zinnfilter

reduzieren die Strahlenbelastung in diesem jungen Patientenkollektiv deutlich [13]. Alternativ dazu haben vollautomatisierte Methoden zum 3‑D-MRT-basierten Volume-Rendering des knöchernen Beckens großes Potenzial, den Einsatz der CT-Bildgebung zu optimieren [14].

## Azetabuläre Minderüberdachung: Dysplasie

Bei der Hüftdysplasie liegen typischerweise eine zu steil gestellte Gelenkpfanne sowie eine defizitäre kraniolaterale Überdachung vor.

### Prävalenz.

Die entwicklungsbedingte Hüftdysplasie wird zumeist im Adoleszentenalter symptomatisch und tritt bei bis zu 20 % der Patienten (v. a. bei Frauen) auf, welche für eine gelenkerhaltenden Hüftoperation abgeklärt werden [6, 15]. Wird die Hüftdysplasie nicht zeitgerecht behandelt, kann sie beim symptomatischen Patienten zur frühzeitigen Coxarthrose führen und ist eine der Hauptgründe für die Implantation einer Hüftprothese bei Patienten unter 60 Jahren [16, 17]. Iatrogen kann eine Dysplasie nach übermäßiger Pfannenrandtrimmung im Rahmen der FAI-Korrektur auftreten und führt zur vorzeitigen Gelenkdegeneration [6, 15].

### Pathomechanismus.

**Figure 4:**
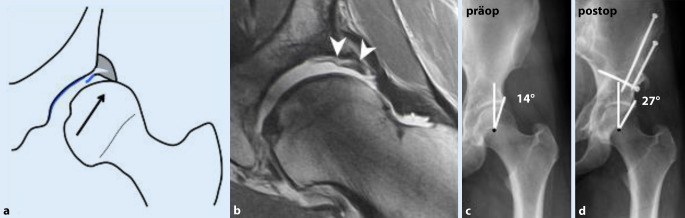

Die insuffiziente azetabuläre Überdachung führt (je nach Dysplasiegrad) zu vermehrter Druck- und Scherbelastung auf Knorpel und Labrum bis hin zur Instabilität (Abb. 4). Dies kann durch eine Coxa valga und erhöhte Femurantetorsion zusätzlich verstärkt werden.

### Diagnostik.

Im Beckenübersichtsröntgen ist der LCE(„lateral center-edge“)-Winkel vermindert (< 23°) und der azetabuläre Index vergrößert (> 13°; Tab. 2; [19]). MR-tomographisches Korrelat der Instabilität ist ein hypertrophiertes, mukoiddegeneriertes Labrum mit Rissbildung und paralabralen Ganglien. In schweren Fällen kommt es auch zu einer Knorpeldelamination, welche sich von zentral nach peripher in das Labrum ausdehnt und dieses schlussendlich vom Knochen abschert (Abb. 4; [18, 20]).

**Table 2:** 

Parameter	Technik	Referenzwerte	Modalität
Alpha-Winkel	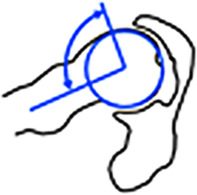	Normal: < 60° Cam-Deformität: > 60°	MRT/CT Axiales Röntgen
CCD(Centrum-Collum-Diaphysen)-Winkel	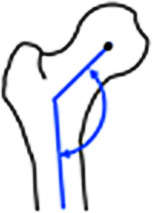	Varus: < 125° Normal: 125–139° Valgus: > 139°	Beckenübersichtsröntgen, MRT/CT
Femurtorsion (Murphy et al.)	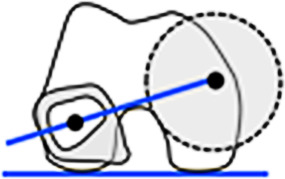	Retrotorsion: < 0° Normal: 10–25° Hohe Antetorsion: > 35°	MRT/CT
LCE(„lateral center-edge“)-Winkel	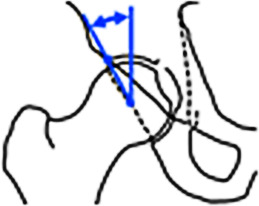	Dysplasie: < 23° Normal: 23–33° Globale Mehrüberdachung: > 39°	Beckenübersichtsröntgen
Azetabulärer Index	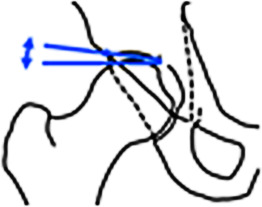	Dysplasie: > 13° Normal: 3–13° Globale Mehrüberdachung: < 0°	Beckenübersichtsröntgen
Protrusio acetabuli	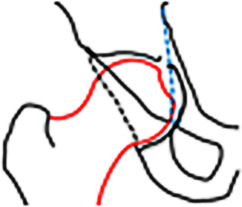	Normal: negativ Globale Mehrüberdachung: positiv	Beckenübersichtsröntgen
„Cross-over sign“	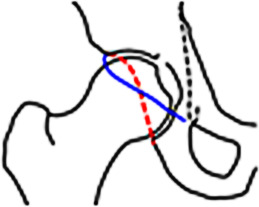	Normal: negativ Retroversion: positiv	Beckenübersichtsröntgen
„Posterior wall sign“	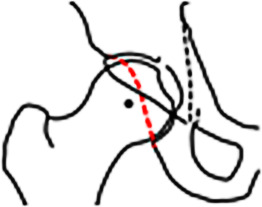	Normal: negativ Retroversion: positiv	Beckenübersichtsröntgen
„Ischial spine sign“	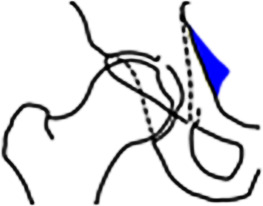	Normal: negativ Retroversion: positiv	Beckenübersichtsröntgen

*MRT* Magnetresonanztomographie, *CT* Computertomographie

### Therapie.

Chirurgische Therapie der Wahl ist die **periazetabuläre Umstellungsosteotomie (PAO)**

Periazetabuläre Umstellungsosteotomie (PAO)

zur Verbesserung der azetabulären Überdachung mit sehr guten langfristigen Ergebnissen im Sinne des Gelenkerhalts [5].

### Merke

Die Hüftdysplasie führt zur Instabilität mit erhöhten Druck- und Scherbelastung auf Knorpel und Labrum.

## Azetabuläres Impaktions-FAI

Das azetabuläre Impaktions-FAI (**Pincer-FAI**

Pincer-FAI)

kann durch eine übermäßige Überdachung oder Fehlorientierung des Azetabulums (d. h. Retroversion) entstehen und verursacht einen harten Anschlag zwischen Azetabulum und Schenkelhals.

### Azetabuläre Mehrüberdachung

#### Prävalenz.

Die azetabuläre Mehrüberdachung hat eine Prävalenz von 15 % bei symptomatischen Patienten und stellt ein Kontinuum aus weniger schweren und schwereren Ausprägungen einer „tiefen Hüfte“ dar [22], welche häufiger bei Frauen auftritt [23]*.*

#### Pathomechanismus.

**Figure 5:**
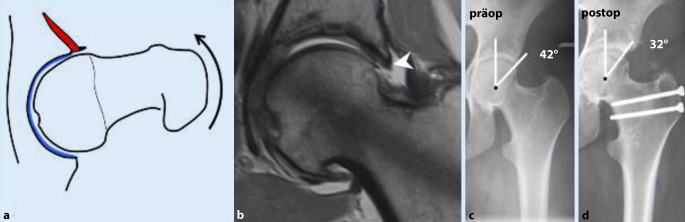

Durch die azetabuläre Mehrüberdachung kommt es in Flexion/Innenrotation zu einem abrupten knöchernen Anschlag (Impaktion) zwischen azetabulärem Rand und Schenkelhals mit Labrumeinklemmung und sekundärer Translation des Femurkopfs inkl. Schäden am posterokaudalen Azetabulum (Abb. 5).

#### Diagnostik.

Ab einem LCE-Winkel von mehr als 33° bzw. mehr als 39° und einem Azetabularindex von weniger als 0° spricht man von Mehrüberdachung respektive **globaler Mehrüberdachung**

(Globale) Mehrüberdachung

(Tab. 2; [22]). Die Extremform ist die **Protrusio acetabuli**

Protrusio acetabuli,

die sich im Beckenübersichtsröntgen im a.p. Strahlengang als Überlappung der Femurkopfkontur mit der ilioischialen Linie darstellt. In der MRT findet sich häufig ein schmales, intrasubstanziell degeneriertes Labrum mit intra-/perilabralen Verknöcherungen (Abb. 5). Der Knorpelschaden beschränkt sich typischerweise auf ein peripheres, semizirkuläres Areal mit ausgedünntem Knorpel. Insbesondere bei schweren Formen der Mehrüberdachung kommt es auch zu einem posterokaudalen Knorpelabrieb (**Contre-coup-Läsion**

Contre-coup-Läsion;

[24]).

#### Therapie.

Je nach Ausprägung der Deformität wird eine umschriebene bis zirkumferenzielle Pfannenrandtrimmung durchgeführt. Jedoch ist die Prognose insbesondere bei Hüften mit globaler (anterior und posterior) Mehrüberdachung deutlich schlechter [25].

### Azetabuläre Mehrüberdachung: Retroversion

#### Prävalenz.

Eine globale (schwere) Retroversion des Azetabulums tritt bei 14 % der symptomatischen Patienten und gehäuft bei Frauen auf [22].

#### Pathomechanismus.

Bei der azetabulären Retroversion handelt es sich um eine Rotationsfehlstellung des Hemipelvis mit Außenrotation der Beckenschaufeln und der Pfanneneingangsebene. Dies prädisponiert zu einem verfrühten, anterioren Impaktions-FAI in Flexion/Innenrotation zwischen azetabulärer Vorderwand und Schenkelhals. Zusätzlich kommt es häufig zu einem extraartikulären Impingement zwischen Spina iliaca anterior inferior (**Spine-Impingement**

Spine-Impingement)

und distalem Schenkelhals, selbst bei normaler Morphologie der Spina iliaca anterior inferior [26].

#### Diagnostik.

Analog zur azetabulären Mehrüberdachung stellt auch die **Retroversion**

Retroversion

ein Kontinuum aus einem umschrieben kranial bis global fehlrotierten Azetabulum dar. Radiographische Zeichen einer azetabulären Retroversion im Beckenübersichtsröntgen im anteroposterioren Strahlengang sind die laterale Projektion der Vorderwand gegenüber der Hinterwand mit Überkreuzen im distalen Verlauf (**„cross-over sign“**

„Cross-over sign“),

eine defizitäre, medial des Femurkopfzentrums verlaufende Hinterwand (**„posterior wall sign“**

„Posterior wall sign“)

und die Projektion der Spina ischiadica (**„ischial spine sign“**

„Ischial spine sign“)

in die Beckeneingangsebene (Tab. 2). Sind diese 3 projektionsradiographischen Zeichen positiv, spricht dies für eine globale azetabuläre Retroversion und somit für ein nach posterior fehlrotiertes, „normal“ großes Azetabulum und ist von einem isolierten positiven „cross-over sign“ zu unterscheiden [27]. In der MRT zeigt sich typischerweise ein normal großes Labrum mit intrasubstanzieller anterosuperiorer Rissbildung.

#### Therapie.

Je nach Schweregrad der Retroversion kann eine Pfannenrandtrimmung ausreichend sein bzw. sollte eine Umstellung der Pfanne mittels antevertierender PAO durchgeführt werden [28].

#### Merke

Das azetabuläre Impaktions-FAI kann durch eine globale Mehrüberdachung oder durch eine Retroversion des Azetabulums entstehen.

## Femorales Inklusions-FAI: Cam-Morphologie

Das Cam-Impingement ist Folge einer aufgehobenen Schenkelhalstaillierung durch einen pathologisch geformten Femurkopf-Schenkelhals-Übergang.

### Prävalenz.

Die Cam-Morphologie ist bei Männern mit Hüftschmerzen mit einer Prävalenz von bis zu 60 % anzutreffen [22]. Unbehandelt kann beim symptomatischen Patienten eine schwere Cam-Deformität bereits mittelfristig zu einer klinisch relevanten Arthrose führen [29].

### Pathomechanismus.

Die Aspherizität des Femurkopfes bzw. die verminderte Schenkelhalstaillierung ermöglicht im Gegensatz zum Impaktions-FAI, wo die Bewegung abrupt gestoppt wird, ein Hineingleiten des Femurs in das Azetabulum mit konsekutiver Induktion von Scherkräften auf die chondrolabrale Übergangszone (Abb. 7), wodurch das Labrum meist basisnah einreißt und der Knorpel sich von peripher nach zentral ablöst (sog. **Teppichphänomen**

„Teppichphänomen“;

[30]).

### Diagnostik.

**Figure 6:**
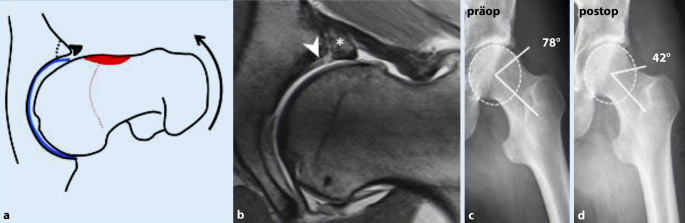

Die Cam-Deformität ist typischerweise ventrokranial lokalisiert, gefolgt von kraniolateral und dorsal [31]. Zur initialen und postoperativen Verlaufsbeurteilung erfolgt die Röntgenbildgebung in 2 Ebenen, d. h. Beckenübersichtsröntgen und axiale Aufnahme. Der Alpha-Winkel (Abb. 6) stellt das klassische Diagnosekriterium einer Cam-Deformität dar, wobei ein Alpha-Winkel von mehr als 60° als Diagnosekriterium verwendet wird (Tab. 2; [2, 32]). In der Praxis ist jedoch eine deskriptive und topographische Beschreibung des Schweregrades und der Ausdehnung der Cam-Deformität entscheidend. Dies erfolgt mittels CT/MRT mit radiären Schichten entlang der Schenkelhalsachse und unter Verwendung des **Zifferblattsystems**

Zifferblattsystem

(Abb. 3). Für den Chirurgen ist die Ausdehnung der Cam-Deformität nach dorsal wichtig, da dort die Korrektur aufgrund der Nähe zu den retinakulären Gefäßen, welche die Durchblutung des Femurkopfes gewährleisten, schwieriger ist [33]. Die Detektion der pathognomonischen azetabulären Knorpeldelamination stellt aufgrund der direkt gegenüberliegenden femoralen Knorpelschicht eine diagnostische Herausforderung dar. Die zusätzliche Verwendung von Traktion in der direkten MR-Arthrographie ermöglicht hingegen eine genauere Darstellung der Knorpeldelamination (Abb. 6; [34, 35]).

### Therapie.

Je nach Ausdehnung erfolgt eine arthroskopische oder offene Schenkelhalstaillierung mit dem Ziel, die physiologische Schenkelhalskontur wiederherzustellen.

### Merke

Die Cam-Deformität befindet sich typischerweise anterosuperior.

## Femorale Fehlstellungen: Torsion und Inklination

### Prävalenz.

Eine zu hohe oder zu niedrige Torsion tritt bei etwa 25 % der symptomatischen Patienten mit FAI oder Dysplasie auf. Eine pathologisch hohe (Coxa valga) oder niedrige (Coxa vara) Schenkelhalsinklination wurde bei je 10 % der Patienten beschrieben [22].

### Pathomechanismus.

Femorale Torsions- und Achsfehler können sowohl Ursache als auch verstärkender Faktor eines FAI sein, je nach assoziierten femoralen/azetebulären Morphologien. Bei Patienten mit einer verminderten femoralen Torsion (**Retrotorsion**

**Figure 7:**
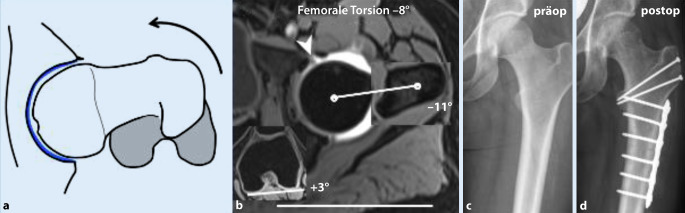

Retrotorsion)

kann es auch bei physiologischer Schenkelhalstaillierung zu einem intra- oder extraartikulären anterioren Impingement in Flexion/Innenrotation kommen (Abb. 7). Eine varische Schenkelhalsachse und/oder zusätzliche Cam‑/Pincer-Deformität kann dies zudem verstärken.

**Figure 8:**
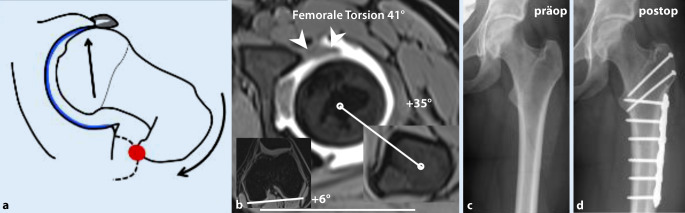

Bei Patienten mit erhöhter femoraler Torsion kann es in Extension/Außenrotation zu einem posterioren extraartikulären Impingement zwischen Trochanter major/minor und Ischium (ischiofemorales Impingement) kommen. Dies führt aufgrund einer sekundären Translation des Femurkopfes nach anterior zu einer dynamischen Instabilität (Abb. 8) und kann die statische Mehrbelastung im Rahmen der Hüftdysplasie weiter verstärken.

### Diagnostik.

Die Schenkelhalsinklination wird mit dem **CCD(Centrum-Collum-Diaphysen)-Winkel**

CCD-Winkel

bestimmt zur Diagnose einer Coxa vara/valga (CCD-Winkel < 125°/> 139°, Referenzwerte Röntgen; Tab. 2; [36]). Die Bestimmung der femoralen Torsion, basierend auf dem konventionellen Röntgen (z. B. mittels **Dunn-Rippstein-Aufnahme**

**Figure 9:**
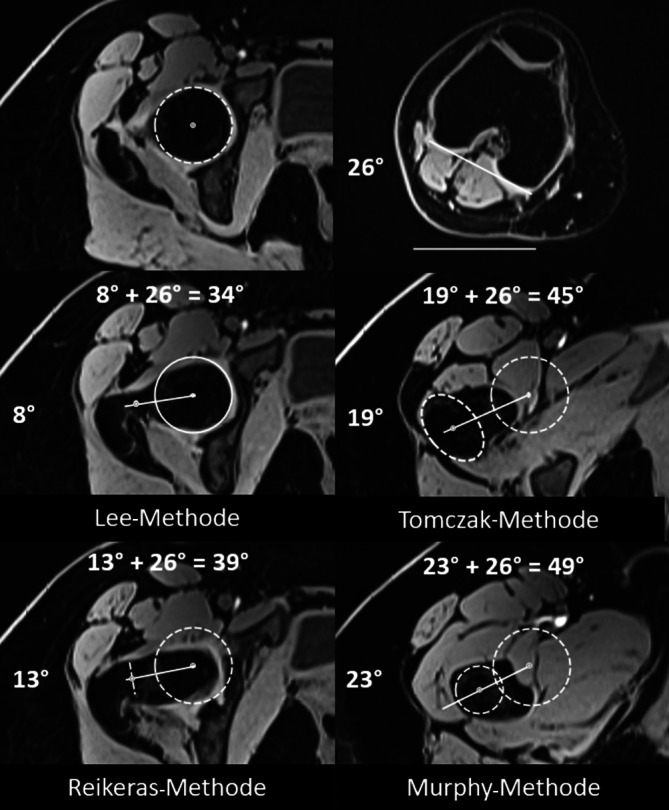

Dunn-Rippstein-Aufnahme),

ist nicht verlässlich und bedarf daher einer Schnittbilddiagnostik. Aufgrund des typischerweise jungen Patientenkollektivs sollte dies idealerweise mittels MRT erfolgen unter Verwendung einer standardisierten Patientenlagerung (in Innenrotation fixierte Füße, keine Flexion der Hüfte durch Lagerungskissen unter dem Knie) und schneller axialer Sequenzen über Becken und Knie [8]. Eine Vielzahl verschiedener Messmethoden wurde in der Literatur beschrieben, die sich, ausgehend vom Femurkopfzentrum, in der Wahl des zweiten proximalen Referenzpunkts unterscheiden (Abb. 9). Je weiter distal dieser Referenzpunkt gesetzt wird, desto höher fallen die gemessenen Werte aus. Diese Unterschiede können beim selben Patienten bis zu 20° betragen; deshalb ist der interdisziplinäre Austausch essenziell, um einen institutionellen Standard zu etablieren [37]. Je nach verwendeter Messmethode gelten andere Referenzwerte. In unserer Institution wird die Methode nach Murphy et al. ([38]; Normalwert: 10–25°; Abb. 9; Tab. 2) verwendet, da sie in Kadaverstudien im Vergleich zur anatomischen Torsion validiert wurde und das Alignment des proximalen Femurs am genauesten berücksichtigt [22, 39]. Für die Messung nach Murphy et al. [38] wird, ausgehend vom Femurrotationszentrum, das Zentrum des Schafts auf Höhe des Trochanter minor als Referenzpunkt verwendet (Abb. 9; Tab. 2).

### Therapie.

Die chirurgische Therapieentscheidung ist abhängig von der gesamten Hüftmorphologie bzw. von begleitenden Hüftdeformitäten, welche vorrangig zu korrigieren sind. In einzelnen Fällen erfolgt eine zusätzliche **femorale Rotationsosteotomie**

Femorale (De‑)Rotationsosteotomie

bzw. Derotationsosteotomie bei Patienten mit Retrotorsion/erhöhter femoraler Antetorsion [40].

### Merke

Die Bestimmung der femoralen Torsion sollte vor jeder Operationsplanung durchgeführt werden.

## Prognostischer Stellenwert der MRT-Bildgebung

**Figure 10:**
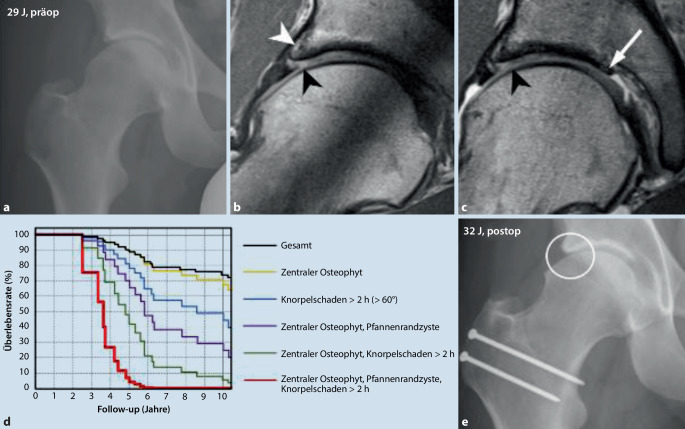

Bereits vorhandene degenerative Veränderungen im Hüftgelenk haben einen negativen Einfluss auf das chirurgische Ergebnis bei gelenkerhaltenden Operationen (Abb. 10; [5, 6]). Da im Röntgen erst fortgeschrittene degenerative Veränderungen sichtbar werden, hat die direkte MR-Arthrographie den höchsten prognostischen Stellenwert hinsichtlich der Detektion degenerativer Veränderungen vor einem gelenkerhaltenden Eingriff. Neben zentralen azetabulären Osteophyten und Pfannenrandzysten stellen Knorpelschäden von mehr als „2 h“ (Verwendung des Zifferblattsystems) den stärksten negativen Prädiktor für den Langzeiterfolg einer FAI-Operation (nach 10 Jahren) dar ([41]; Abb. 10). Neben diesen morphologischen Prädiktoren haben biochemische Knorpelmappingmethoden, die eine quantitative Bestimmung der Knorpelqualität erlauben **(„delayed gadolinium enhanced magnetic resonance imaging of cartilage“, dGEMRIC**

dGEMRIC („delayed gadolinium enhanced magnetic resonance imaging of cartilage“)),

prognostisches Potenzial und wurden z. B. als verlässliche negative Prädiktoren für den Langzeiterfolg nach PAO bei Patienten mit Dysplasie bei 1,5 T beschrieben [42, 43]. Die zeitaufwändige manuelle Auswertung, stärkere Magnetfeldinhomogenitäten bei 3 T und die fehlende Gelenkdistension bei i.v. Kontrastmittelapplikation haben eine standardmäßige Anwendung des **Knorpelmappings**

Knorpelmapping

im klinischen Alltag bisher verhindert. Fortschritte im Bereich des **Deep Learning**

Deep Learning

zur automatischen 3‑D-Segmentation und Knorpelanalyse anhand robusterer T1-Mapping- sowie neuere native T2/T1-rho-Mapping-Sequenzen stellen vielversprechende Ansätze zur verbesserten präoperativen Schadenanalyse [44, 45] und möglichen Integration in den klinischen Alltag dar.

### Merke

Die direkte MR-Arthrographie hat den höchsten prognostischen Stellenwert hinsichtlich des Langzeiterfolgs eines hüftgelenkerhaltenden Eingriffs.

## Fazit für die Praxis

- Grundlage der diagnostischen Abklärung vor gelenkerhaltenden Eingriffen an der Hüfte sind die Projektionsradiographie in 2 Ebenen zur Beurteilung der azetabulären Überdachung und Version sowie die initiale Beurteilung des Arthrosegrades.
- Ein dezidiertes MRT-Protokoll sollte flüssigkeitssensitive Sequenzen des Beckens zum groben Ausschluss von Begleitpathologien, axiale Sequenzen zur Bestimmung der Femurtorsion sowie hüftzentrierte, multiplanare (inkl. radiärer Schichten) Sequenzen zur Detektion chondrolabraler Schäden beinhalten.
- Die direkte MR-Arthrographie stellt die Methode der Wahl zur Detektion von chondrolabralen Schäden dar.
- Hauptpathomechanismen der Hüfte sind das Impingement und die Dysplasie, welche auch in Kombination auftreten können. Je nach Kombination können sich diese aggravieren oder kompensieren.
- Femorale Torsionspathologien stellen neben der Cam- und der Pincer-Morphologie den dritten Pfeiler des femoroazetabulären Impingements dar.

## CME-Fragebogen

Welche Modalität/Schichtorientierung stellt den Schenkelhalsübergang zur Beurteilung einer Cam-Deformität am genauesten dar?

Beckenübersichtsröntgen

Ultraschall

Lauenstein-Aufnahme

Radiäre Magnetresonanztomographie (MRT)

„Axial oblique“ MRT

Welches Röntgenzeichen ist *nicht* mit einer Pincer-Morphologie assoziiert?

„Cross-over sign“

„Ischial spine sign“

„Posterior wall sign“

Femorale Retrotorsion

Protrusio acetabuli

Welches degenerative Zeichen in einer Magnetresonanztomographie der Hüfte stellt den stärksten negativen Prädiktor für ein gutes klinisches Ergebnis nach einer FAI(femoroazetabuläres Impingement)-Operation dar?

Labrumläsion

„Herniation pit“

Pfannenrandosteophyt

Pfannenrandzyste

Knorpelschaden > 2 h

Ein 28-Jähriger Patient mit Hüftschmerzen wird vom Orthopäden zur MRT der Hüfte zugewiesen mit Frage nach Hüftimpingement. Welche Sequenz muss das MRT-Protokoll NICHT zwingend beinhalten?

Flüssigkeitssensitive Sequenz des Beckens

Axiale Sequenzen des distalen Femurs

Bilder in radiärer Orientierung um den Schenkelhals

Multiplanare (coronale, sagittale, axiale, axial-oblique) Sequenzen

T1w Sequenz des Beckens

Welche der nachfolgenden Morphologien prädisponiert zu einem posterioren extraartikulären Impingement?

Exzessiv erhöhte femorale Antetorsion

Cam-Deformität

Azetabuläre Retroversion

Hüftdysplasie

Femorale Retrotorsion

Zur Bestimmung der femoralen Torsion wurde welche Methode mit Kadaverstudien validiert und berücksichtigt das Alignement des proximalen Femurs am genauesten?

Lee

Tomczak

Reikeras

Murphy

Jarrett

Wo befindet sich typischerweise die Cam-Deformität?

Anterosuperiores Azetabulum

Anterosuperiorer Schenkelhals

Posteroinferiorer Schenkelhals

Superolateraler Schenkelhals

Spina iliaca anterior inferior

Welche der nachfolgend genannten Techniken bildet die Basis der Diagnostik bei gelenkerhaltenden Hüfteingriffen?

Computertomographie

Magnetresonanztomographie

Röntgen

Ultraschall

EOS

Welche Hüftdeformität kann ein anteriores impingement durch eine Retroversion des Azetabulums kompensieren?

Azetabuläre Mehrüberdachung

Erhöhte femorale Antetorsion

Coxa vara

Cam Deformität

Coxa valga

Welche der folgenden Techniken ist die Methode der Wahl zur Detektion von chondrolabralen Schäden im Hüftgelenk?

Computertomographie-Arthrographie

Direkte MR-Arthrographie

Ultraschall

Native MR-Tomographie des Beckens

Indirekte MR-Arthographie
